# Supplementary material for: Bayesian efficient coding
Source: PLoS Comput Biol. 2026 Nov 4;22(11):e1014894. doi: 10.1371/journal.pcbi.1014894 (PMC13747246; doi:10.1371/journal.pcbi.1014894)
Supplement: Supplementary file 1 [file pcbi.1014894.s001.pdf]

S1 Appendix. Supporting information

Loss functionals

Loss functionals quantify the goodness of the posterior distribution $P(\mathbf{x} \,|\, \mathbf{y}, \theta)$. There are broadly two classes of loss functionals: **entropic loss** or **reconstruction loss**. Entropic losses quantify the average uncertainty of the posterior: The more concentrated the posterior, the better.

$$L(P(\mathbf{x} \,|\, \mathbf{y})) = \begin{cases} \mathbb{E}_{\mathbf{x}|\mathbf{y}} \left[-\log P(\mathbf{x}|\mathbf{y}) \right] & \text{(S1a)} \\ \qquad \text{(Shannon entropy)} & \\ \dfrac{1}{1-\alpha} \log \mathbb{E}_{\mathbf{x}|\mathbf{y}} \left[P(\mathbf{x}|\mathbf{y})^{\alpha-1} \right] & \text{(S1b)} \\ \qquad \text{(Rényi } \alpha\text{-entropy)} & \\ \dfrac{1}{1-\alpha} \mathbb{E}_{\mathbf{x}|\mathbf{y}} \left[P(\mathbf{x}|\mathbf{y})^{\alpha-1} - 1 \right] & \text{(S1c)} \\ \qquad \text{(Tsallis entropy)} & \\ \mathrm{Tr} \left(\mathrm{cov} \left[\mathbf{x} \,|\, \mathbf{y} \right]^{\frac{p}{2}} \right) & \text{(S1d)} \\ \qquad (p\text{-covtropy}) & \end{cases}$$

Both eq. S1b and eq. S1c converge to eq. S1a in the limit of $\alpha \to 1$.

Traditional entropic losses do not discriminate points in the stimulus domain, that is, any error is treated equally. However, when stimulus space is $\mathbb{R}^n$, it makes sense to prefer locally tight posterior distributions. One such measure is the p-covtropy which measures the posterior concentration around the posterior mean (only defined for distributions in $\mathbb{R}^d$). Note that $\frac{p}{2}$'th power of the posterior covariance matrix corresponds to taking p'th power of the standard deviations along each principal axis through diagonalization.

The second class of loss functionals depends on a specific readout (a.k.a. estimator or reconstruction) $\hat{\mathbf{x}}(\mathbf{y})$ that maps the neural response $\mathbf{y}$ back to the stimulus domain. We consider a general average reconstruction error loss functional of the form: $L(P(\mathbf{x} \,|\, \mathbf{y})) = \mathbb{E}_{\mathbf{x}|\mathbf{y}} \left[d(\mathbf{x}, \hat{\mathbf{x}}(\mathbf{y})) \right]$ where $d(\cdot, \cdot)$ is a distortion measure.

$$L(P(\mathbf{x} \,|\, \mathbf{y})) = \begin{cases} \mathbb{E}_{\mathbf{x}|\mathbf{y}} \|\mathbf{x} - \hat{\mathbf{x}}(\mathbf{y})\|_p & \text{(S2a)} \\ \qquad (p\text{-norm error}) & \\ \mathbb{E}_{\mathbf{x}|\mathbf{y}} \|\mathbf{x} - \hat{\mathbf{x}}(\mathbf{y})\|_2^2 & \text{(S2b)} \\ \qquad \text{(mean squared error)} & \\ \mathbb{E}_{\mathbf{x}|\mathbf{y}} \left[1 - \delta(\mathbf{x} - \hat{\mathbf{x}}(\mathbf{y})) \right] & \text{(S2c)} \\ \quad = P(\mathbf{x} \neq \hat{\mathbf{x}}(\mathbf{y}) | \mathbf{y}) \qquad \text{(0-1 loss)} & \end{cases}$$

Mean squared error and the 0-1 loss are widely used in communication theory, machine learning, and statistics in general.

There are loose bounds that connect the posterior entropy with Bayesian error rate (e.g. Fano bound for classification) [1–3]. However, these bounds do not justify infomax strategy because better Bayesian error rate can be achieved with a system that directly optimizes for the target error measure.

Note the similarity between p-norm error and the p-covtropy when posterior mean is used for decoding. They coincide when $p = 2$, however for $p \neq 2$, unlike the p-norm error, p-covtropy is invariant under unitary transformation of the stimulus space (which is the key difference between the 2D Gaussian and the linear receptive field examples). This is an important distinction if axes in $\mathbf{x}$ do not have special meaning, and rotated

posteriors are considered equally good. We can make this connection rigorous for a Gaussian posterior, $\mathbf{x} \,|\, \mathbf{y} \sim \mathcal{N}(\mu, C)$. Let $C = UDU^{-1}$ be the eigendecomposition of the covariance matrix. Let $Z \sim \mathcal{N}(0, D)$ be aligned on the principal axes, the p-th power of the p-norm is given by,

$$\begin{aligned}\mathbb{E}_Z\left[\sum_i |z_i|^p\right] &= \sum_i \mathbb{E}_Z\left[|z_i|^p\right] = \sum_i \kappa(p)\sigma_i^p \\ &= \kappa(p)\,\mathrm{Tr}\left(D^{\frac{p}{2}}\right) = \kappa(p)\,\mathrm{Tr}\left(C^{\frac{p}{2}}\right)\end{aligned}$$

where $\kappa(p) = \frac{1}{\sqrt{\pi}} 2^{\frac{p}{2}} \Gamma\left(\frac{p+1}{2}\right)$.

In Linear receptive fields, we discuss the loss functions only for Gaussian posteriors. However, covtropy is well defined for any distribution with a valid covariance matrix. Note that minimizing the limiting case of covtropy for $p \to 0$ and maximizing mutual information do not coincide in general since posterior entropy is not a sole function of the covariance in general.

Equivalence of MSE and covtropy for $p = 2$

Here we provide a simple proof showing that minimizing covtropy for $p = 2$ corresponds to minimizing mean squared error (MSE). Let $\mathbb{E}[\|\mathbf{x} - \hat{\mathbf{x}}(\mathbf{y})\|_2^2]$ denote the MSE for any estimator $\hat{\mathbf{x}}(\mathbf{y})$, where expectation is taken with respect to the posterior $P(\mathbf{x}|\mathbf{y})$. It is well known that the posterior mean or "Bayes least squares" estimator $\hat{\mathbf{x}}_{BLS} = \mathbb{E}[\mathbf{x}|\mathbf{y}]$ achieves the minimum of the MSE, which is then given by $\mathbb{E}[\|\mathbf{x} - \hat{\mathbf{x}}_{BLS}\|_2^2] = \mathbb{E}[(\mathbf{x} - \hat{\mathbf{x}}_{BLS})^\top(\mathbf{x} - \hat{\mathbf{x}}_{BLS})]$. We can use identities involving trace and the definition of covariance to show that MSE is equal to $\mathbb{E}[\mathrm{Tr}[(\mathbf{x} - \hat{\mathbf{x}}_{BLS})^\top(\mathbf{x} - \hat{\mathbf{x}}_{BLS})]] = \mathbb{E}[\mathrm{Tr}[(\mathbf{x} - \hat{\mathbf{x}}_{BLS})(\mathbf{x} - \hat{\mathbf{x}}_{BLS})^\top]] = \mathrm{Tr}[\mathbb{E}[(\mathbf{x} - \hat{\mathbf{x}}_{BLS})(\mathbf{x} - \hat{\mathbf{x}}_{BLS})^\top]] = \mathrm{Tr}[\Sigma]$, which is the covtropy with $p = 2$. Thus, the code that achieves minimum $p = 2$ covtropy corresponds to the code that achieves minimum MSE point estimation of the stimulus.

Linear receptive fields

Here we derive the optimal receptive fields for linear encoding under Gaussian noise (see Linear receptive fields), among encoders that share an eigenbasis with Q and R; this is no restriction when R is proportional to the identity. Recall the model (eqs. 3-5):

$$\mathbf{x} \sim \mathcal{N}(0, Q); \tag{S3}$$

$$\mathbf{y} = W\mathbf{x} + \epsilon, \qquad \epsilon \sim \mathcal{N}(0, R), \tag{S4}$$

and we wish to find the optimal W subject to the power constraint

$$\mathbb{E}[\mathbf{y}^\top \mathbf{y}] = \mathrm{Tr}[WQW^\top + R] \leq c. \tag{S5}$$

We make the assumption that Q,R, and W matrices commute, which means they share a common eigenbasis or can be diagonalized by the same orthogonal matrix. This occurs, for example, if all three are circulant matrices, so that W consists of shifted copies of a single RF shape.

For Fig 4, Q is the 100-dimensional projection of a $1/f^2$ stimulus spectrum and $R = 0.001I$, giving irreducible noise power $\mathrm{Tr}(R) = 0.1$. The high-, intermediate-, and low-power conditions use $c = 0.655\mathrm{Tr}(Q) = 99.5643$, 0.5, and 0.2, respectively. The all-active closed form in the main text applies while the matrix inside its square root is positive semidefinite. At lower power, the constrained optimum sets some gains to zero

and reallocates power across the remaining modes. At intermediate power, the infomax and $p = 1, 2, 4$ encoders retain 100, 100, 100, and 57 active modes; at low power they retain 100, 76, 38, and 23.

Infomax

Maximizing information is equivalent to minimizing the posterior entropy:

$$\begin{aligned} L = H(\mathbf{x}|\mathbf{y}, \theta) &= \tfrac{1}{2} \log |2\pi e \Sigma| \\ &= -\tfrac{1}{2} \log |W^\top R^{-1} W + Q^{-1}| + const. \end{aligned} \tag{S6}$$

We can find the optimal W subject to the power constraint above using the method of Lagrange multipliers:

$$\begin{aligned} &\frac{\partial}{\partial W}(L + \lambda \mathrm{Tr}[WQW^\top + R]) \\ &= -\tfrac{1}{2}(W^2 R^{-1} + Q^{-1})^{-1} 2WR^{-1} + 2\lambda WQ = 0, \end{aligned} \tag{S7}$$

where λ is a Lagrange multiplier. This implies

$$(W^2 R^{-1} + Q^{-1}) RQ = \tfrac{1}{2\lambda} I, \tag{S8}$$

giving

$$W^2 Q + R = \tfrac{1}{2\lambda} I, \tag{S9}$$

and finally

$$W = (\tfrac{1}{2\lambda} I - R)^{\frac{1}{2}} Q^{-\frac{1}{2}}. \tag{S10}$$

Plugging this solution into the constraint (eq. S5) gives

$$\mathrm{Tr}[\tfrac{1}{2\lambda} I] \leq c \tag{S11}$$

which implies $1/(2\lambda) = c/n$, where $n = \dim(\mathbf{y})$ is the number of neurons. Substituting for λ gives the desired expression:

$$\hat{W}_{\mathrm{infomax}} = (\tfrac{c}{n} I - R)^{\frac{1}{2}} Q^{-\frac{1}{2}}. \tag{S12}$$

Minimum p-Covtropy

We can take a similar approach for the loss function we have called p-covtropy, which is effectively the mean p'th power error in the eigenbasis of the posterior distribution. This is given by

$$L_p = \mathrm{Tr}[\Sigma^{\frac{p}{2}}], \tag{S13}$$

which involves the posterior covariance matrix Σ to the matrix-power $p/2$. It turns out this loss function has the same optimum as infomax loss in the limit $p \to 0$.

Once again, the method of Lagrange multipliers allows us to solve for W explicitly in the case that W, R, and Q commute. Taking the derivative of the Lagrangian with respect to W and setting to zero yields

$$\begin{aligned} \frac{\partial}{\partial W}\left(L + \lambda \mathrm{Tr}\big[\mathrm{cov}(\mathbf{y})\big]\right) &= \frac{\partial}{\partial W}(\mathrm{Tr}[\Sigma^{\frac{p}{2}}] + \lambda \mathrm{Tr}[WQW^\top + R]) \\ &= -p(W^2 R^{-1} + Q^{-1})^{-\frac{p}{2}-1} WR^{-1} + 2\lambda WQ = 0. \end{aligned}$$

This can be simplified to

$$(W^2 R^{-1} + Q^{-1})^{-\frac{p+2}{2}} = \tfrac{2\lambda}{p} QR$$

and so

$$W^2 = R\left(\tfrac{2\lambda}{p}QR\right)^{\frac{-2}{p+2}} - RQ^{-1} \tag{S14}$$

$$= \left((\tfrac{p}{2\lambda})^{\frac{2}{p+2}}(QR)^{\frac{p}{p+2}} - R\right)Q^{-1} \tag{S15}$$

and finally

$$\hat{W}_p = \left(\alpha(QR)^{\frac{p}{p+2}} - R\right)^{\frac{1}{2}} Q^{-\frac{1}{2}}, \tag{S16}$$

with $\alpha = (\frac{p}{2\lambda})^{\frac{2}{p+2}}$. Substituting $\hat{W}_p$ into the power constraint (eq. S5) gives:

$$\mathrm{Tr}[\alpha(QR)^{\frac{p}{p+2}}] \leq c, \tag{S17}$$

which achieves the constraint with equality when

$$\alpha = \frac{c}{\mathrm{Tr}[(QR)^{\frac{p}{p+2}}]}, \tag{S18}$$

which completes the derivation.

Closed-form optimal nonlinearity for a log-logistic prior

When the stimulus prior is log-logistic, which the Naka-Rushton family fit to Laughlin's contrast distribution is (Methods), the optimum can instead be written down in closed form in the limit of many response levels. The exponent p then enters only through the two parameters of a beta distribution, and a solution exists only for p below the tail index of the prior.

Let g be an increasing nonlinearity of the Weber contrast $x \geq -1$, mapping it onto the response range normalized to $[0, 1]$, so that $g(-1) = 0$ and $g(\infty) = 1$. Its derivative $\rho = g'$ is a probability density on the stimulus axis: $\rho(x)$ is the fraction of the response range spent per unit stimulus near x, so it sets the local resolution of the encoder, and $\int \rho = 1$ is the range constraint itself.

In the high-resolution limit, where precision is limited by a large but finite number of response levels, the local cell width is proportional to $1/\rho(x)$, and the average reconstruction error (eq. 16) takes the form $L^p \propto \int P(x)\,\rho(x)^{-p}\,\mathrm{d}x$ to leading order. Minimizing it subject to $\int \rho = 1$ gives the classical optimal point density of high-resolution quantization [4–6], with the asymptotic theory for a prior on an unbounded support due to Zador [7]:

$$\rho_p^\star \propto P^s, \qquad g_p^\star(x) = \frac{\int_{-1}^{x} P(t)^s\,\mathrm{d}t}{\int_{-1}^{\infty} P(t)^s\,\mathrm{d}t}, \qquad s := \frac{1}{1+p}. \tag{S19}$$

The case $p = 2$, $\rho^\star \propto P^{1/3}$, is the Panter-Dite rule [5]. Raising p flattens the resolution profile, moving resolution away from the mode of the prior and into the tails, where errors are large.

It is convenient to describe a nonlinearity by how it warps the uniformized stimulus $u = F(x)$, which is uniform on $[0, 1]$, where F is the prior CDF. Every increasing g with $g(-1) = 0$ and $g(\infty) = 1$ can be written as $g = W \circ F$ for a single increasing warp $W : [0, 1] \to [0, 1]$ with $W(0) = 0$ and $W(1) = 1$. The Naka-Rushton CDF fit to the contrast distribution (Methods), with its location at the contrast floor, $a = -1$, is exactly a log-logistic prior: $\log(1 + x)$ then has a logistic distribution with shape b, and in the uniformized coordinate the density is a product of powers of u and $1 - u$,

$$F(x) = \frac{(1+x)^b}{(1+x)^b + c}, \qquad P(x) = \frac{b\,c\,(1+x)^{b-1}}{\left((1+x)^b + c\right)^2}, \tag{S20}$$

with b and c the fitted Naka-Rushton exponent and semisaturation, and

$$\phi(u) := P\big(F^{-1}(u)\big) = b\, c^{-1/b}\, u^{1-1/b}\, (1-u)^{1+1/b}. \tag{S21}$$

Under the change of variables $u = F(x)$, so that $\mathrm{d}u = P(x)\,\mathrm{d}x$,

$$P(x)^s\,\mathrm{d}x = \phi(u)^{s-1}\,\mathrm{d}u \propto u^{A_p-1}(1-u)^{B_p-1}\,\mathrm{d}u, \qquad A_p = \frac{1+p/b}{1+p}, \quad B_p = \frac{1-p/b}{1+p}.$$

Thus, the integrals in (eq. S19) are incomplete beta integrals. The optimal warp is therefore the CDF of a beta distribution,

$$g_p^\star = W_p \circ F, \qquad W_p(u) = I_u(A_p, B_p)\,, \tag{S22}$$

where $I_u(A, B)$ is the regularized incomplete beta function, that is, the CDF of $\mathrm{Beta}(A, B)$ evaluated at u. The entire dependence on p is carried by the two numbers A_p and B_p (panels A and B of Fig A).

The same two numbers determine the response distribution. The response is $y = W_p(u)$ with u uniform, so its density is $1/w_p\big(W_p^{-1}(y)\big)$, where w_p is the $\mathrm{Beta}(A_p, B_p)$ density. Here $A_p < 1$ exactly when $b > 1$, that is, when the prior has an interior mode, while $0 < B_p < 1$ for every $0 < p < b$; with both parameters below 1 the beta density is U-shaped, so $P(y)$ is peaked at intermediate response levels (panel C of Fig A, and Fig 5C). Its peak sits where w_p is smallest, at $u = (b-1)/(2b)$, which is the prior CDF evaluated at the mode of the prior. For every $p > 0$, the most frequently used responses therefore encode the most probable stimulus.

They also give both ends of the family. Since $A_0 = B_0 = 1$ and $\mathrm{Beta}(1, 1)$ is uniform, W_0 is the identity and $g_0^\star = F$, which is histogram equalization: infomax is the $p \to 0$ member of this family rather than a separate criterion [8, 9]. At the other end, beta normalization requires $A_p > 0$ and $B_p > 0$. The first holds for every $p > 0$, whereas the second holds only for $p < b$. This is also the moment condition on the prior: since $P(x)$ falls as $x^{-(b+1)}$, $\mathbb{E}|x|^p < \infty$ exactly when $p < b$. Thus, *no bounded-range code has finite L_p error for $p \geq b$.* As p approaches b from below, the optimum spends an ever larger share of its response range on stimuli beyond any fixed contrast (Fig A).

The Naka-Rushton fit quoted in Methods has a free location $a \leq -1$ rather than $a = -1$, and the physical contrast floor then truncates it. Writing F_0 for the untruncated fit, which is log-logistic in $x - a$ with the same shape b, the prior is

$$F_0(x) = \frac{|x-a|^b}{|x-a|^b + c}, \qquad F(x) = \frac{F_0(x) - F_0(-1)}{1 - F_0(-1)}, \qquad x \geq -1. \tag{S23}$$

Truncation multiplies P by a constant on the support, which cancels in (eq. S19), so A_p and B_p are unchanged and the only difference is an affine rescaling of the same warp,

$$g_p^\star(x) = \frac{W_p\big(F_0(x)\big) - m}{1 - m}, \qquad m := W_p\big(F_0(-1)\big). \tag{S24}$$

The case $a = -1$ has $F_0(-1) = 0$ and $m = 0$, which returns eq. S22. Fig A shows this family for the fitted prior.

The closed form above is the high-resolution ($N \to \infty$) limit of the exact finite-N quantizer problem it was derived from. Fig B checks it directly against that finite-N problem, solved independently by Lloyd-Max fixed-point iteration [10, 11] rather than by the formula being checked.

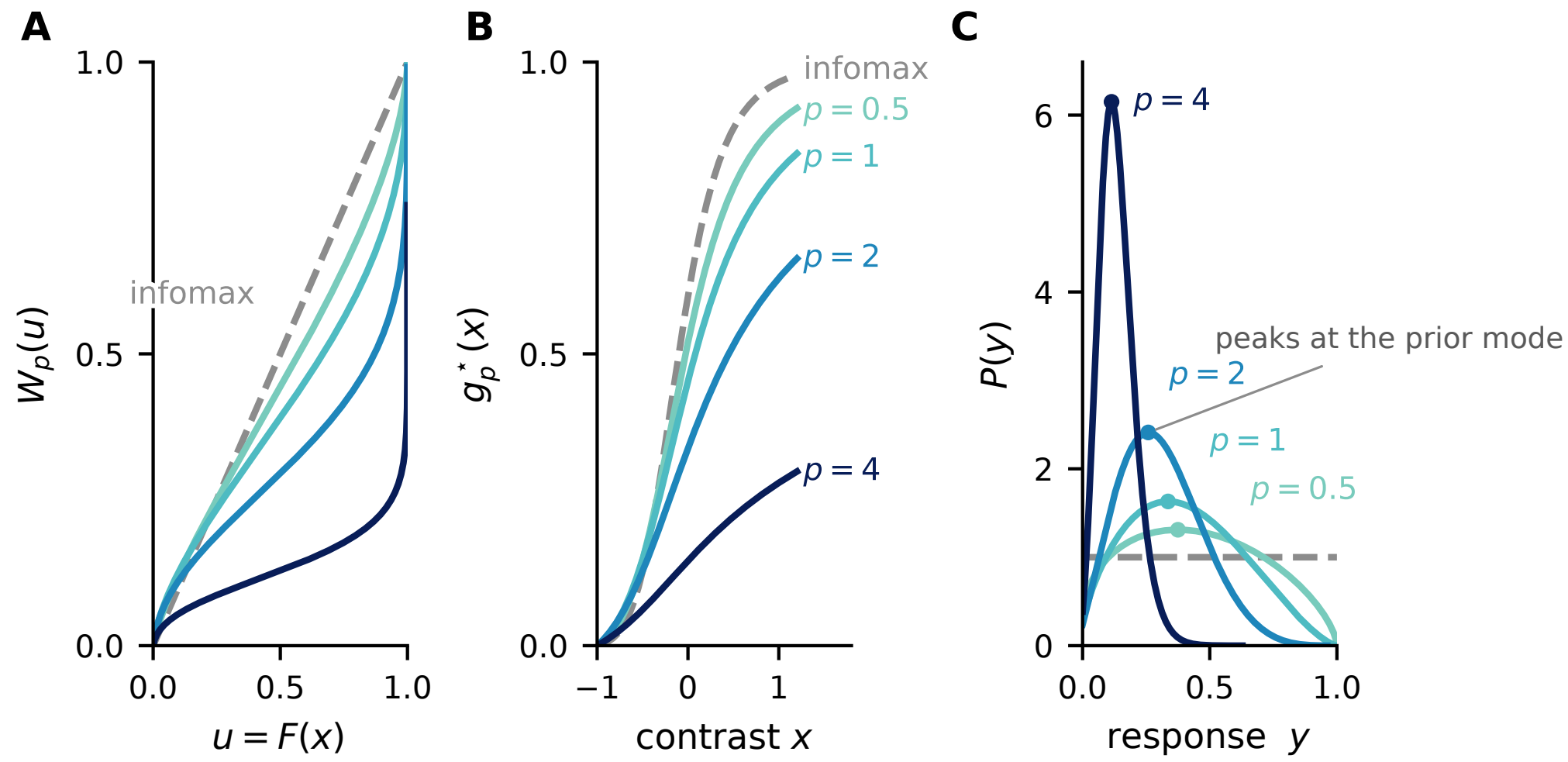

Fig A. Increasing p shifts coding resolution from common contrasts toward the tails of the stimulus distribution. **(A)** Optimal warps W_p of the uniformized stimulus $u = F(x)$ for the fitted contrast prior. The identity warp at $p = 0$ is the infomax solution; increasing p assigns more of the response range to rare, large contrasts. **(B)** The same solutions in contrast coordinates, $g_p^\star = W_p \circ F$. **(C)** Response distributions induced by each code. Infomax uses every response equally often, whereas each $p > 0$ solution is peaked at the response assigned to the most probable stimulus. The largest displayed exponent shows the limiting behavior as p approaches the tail index b, beyond which no finite-error solution exists.

Numerical optimization and evaluation for the LNP model

The LNP optima of Fig 6B have no closed form. Let x denote the STA-projected stimulus and $g(x)$ the conditional mean spike count. For each count y, we use the exact Poisson posterior $P(x \mid y) \propto \text{Poisson}(y; g(x))\, P(x)$ and its Bayes estimator under L_p loss. We minimize the response-averaged reconstruction error,

$$D_p(g) \;=\; \sum_y P(y)\, \min_{\hat{x}} \mathbb{E}[\,|x - \hat{x}|^p \mid y\,]\,, \qquad \text{subject to } \mathbb{E}[y] = \hat{c}. \tag{S25}$$

Here $\hat{c}$ is the empirical mean count. We optimize over non-decreasing, non-negative g from multiple starts.

The mean-rate optimum has zero baseline, whereas H1 fires spontaneously. Before comparing shapes, we therefore fit the affine map $\lambda(x) = a\, g(x) + b$ by Poisson maximum likelihood. Let $\hat{\mu}(x)$ and $\hat{v}(x)$ denote the posterior mean and variance of the log rate under the Gaussian-process-Poisson fit (Blowfly H1 data from [12]). We compare the transported model with this fit using

$$\chi^2 \;=\; \left\langle \left(\frac{\log(a\, g(x) + b) - \hat{\mu}(x)}{\sqrt{\hat{v}(x)}} \right)^2 \right\rangle, \tag{S26}$$

where the brackets denote an average over the data-constrained part of the measured curve.

The normalized STA projection x is approximately standard normal in this experiment, so a standard-Gaussian prior matches the laboratory stimulus. The best-fitting standard-Gaussian solution nevertheless overpredicts H1 at large positive projections. Natural-flight yaw velocities have a sharp central peak and long tails produced by intersaccadic and saccadic motion [13], and previous work modeled this same H1 recording with a power-law speed prior [14]. These results motivate, but do not determine, broader or heavier-tailed adaptation priors for the STA projection. We

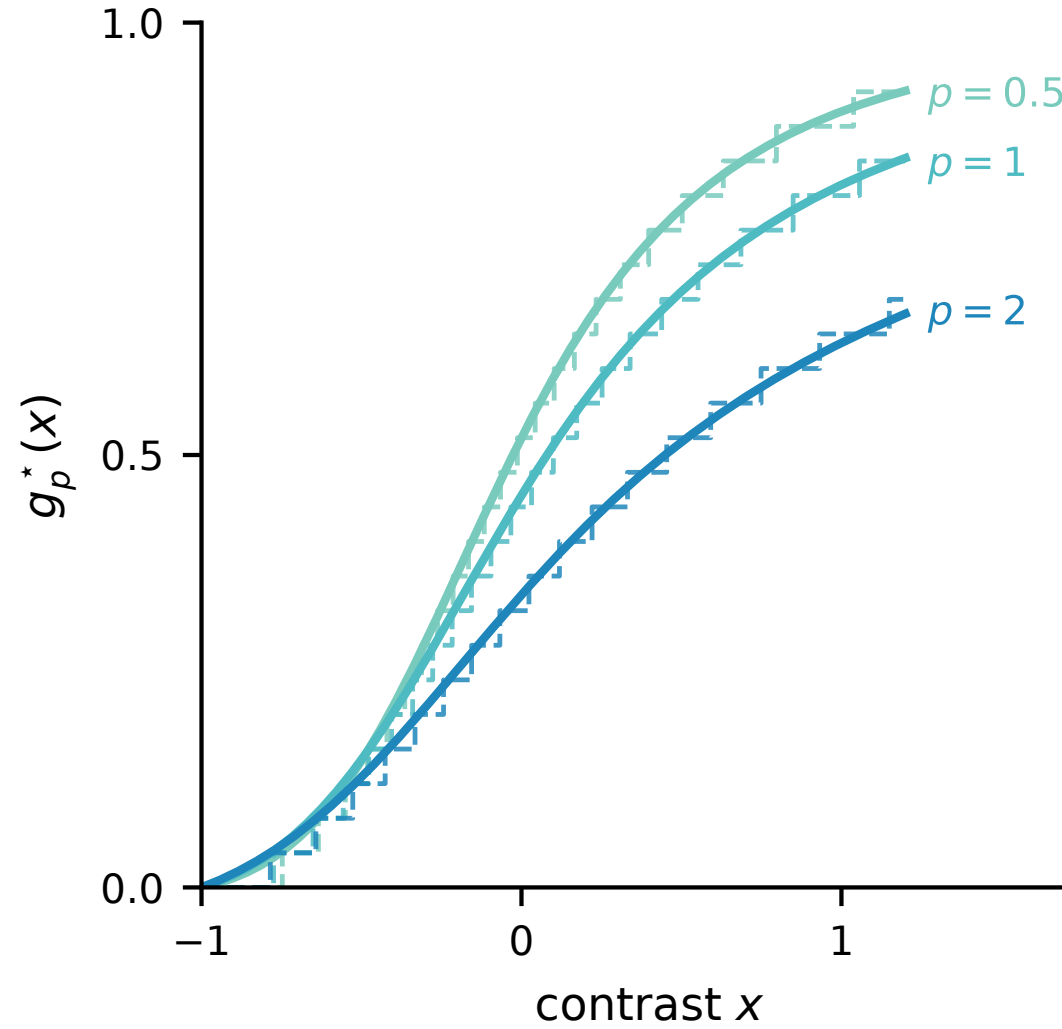

Fig B. The closed-form nonlinearities agree with independently optimized finite quantizers. The high-resolution solution $g_p^\star(x)$ (solid) is compared with an independently optimized finite-level Lloyd-Max quantizer for the same prior and exponent (dashed staircase). Their close agreement shows that the closed form describes the optimal code well at finite response resolution.

therefore vary Gaussian width and Student-t tail index (Fig D). The Student-t family was scaled to match the interquartile range of the standard normal, isolating tail shape from central scale. Across both families, p changes the predicted nonlinearity more than the prior parameter, and the best matches remain far from the infomax limit.

A Student-t prior has no finite p-th moment for $p \geq \nu$, so those solutions depend on restricting the stimulus range to that supported by the data. We display $\nu = 1.5$ because it is heavy-tailed but places less mass outside this range than the more extreme priors. The improved match under heavy tails therefore does not establish that H1 is adapted to a heavy-tailed natural ensemble.

References

1. Seidler J. Bounds on the mean-square error and the quality of domain decisions based on mutual information. Information Theory, IEEE Transactions on. 1971;17(6):655–665.

2. Feder M, Merhav N. Relations between entropy and error probability. IEEE transactions on information theory / Professional Technical Group on Information Theory. 1994;40(1):259–266.

3. Thomson EE, Kristan WB. Quantifying stimulus discriminability: a comparison of information theory and ideal observer analysis. Neural computation. 2005;17(4):741–778.

4. Bennett WR. Spectra of quantized signals. The Bell System Technical Journal. 1948;27(3):446–472. doi:10.1002/j.1538-7305.1948.tb01340.x.

5. Panter PF, Dite W. Quantization distortion in pulse-count modulation with nonuniform spacing of levels. Proceedings of the IRE. 1951;39(1):44–48. doi:10.1109/jrproc.1951.230419.

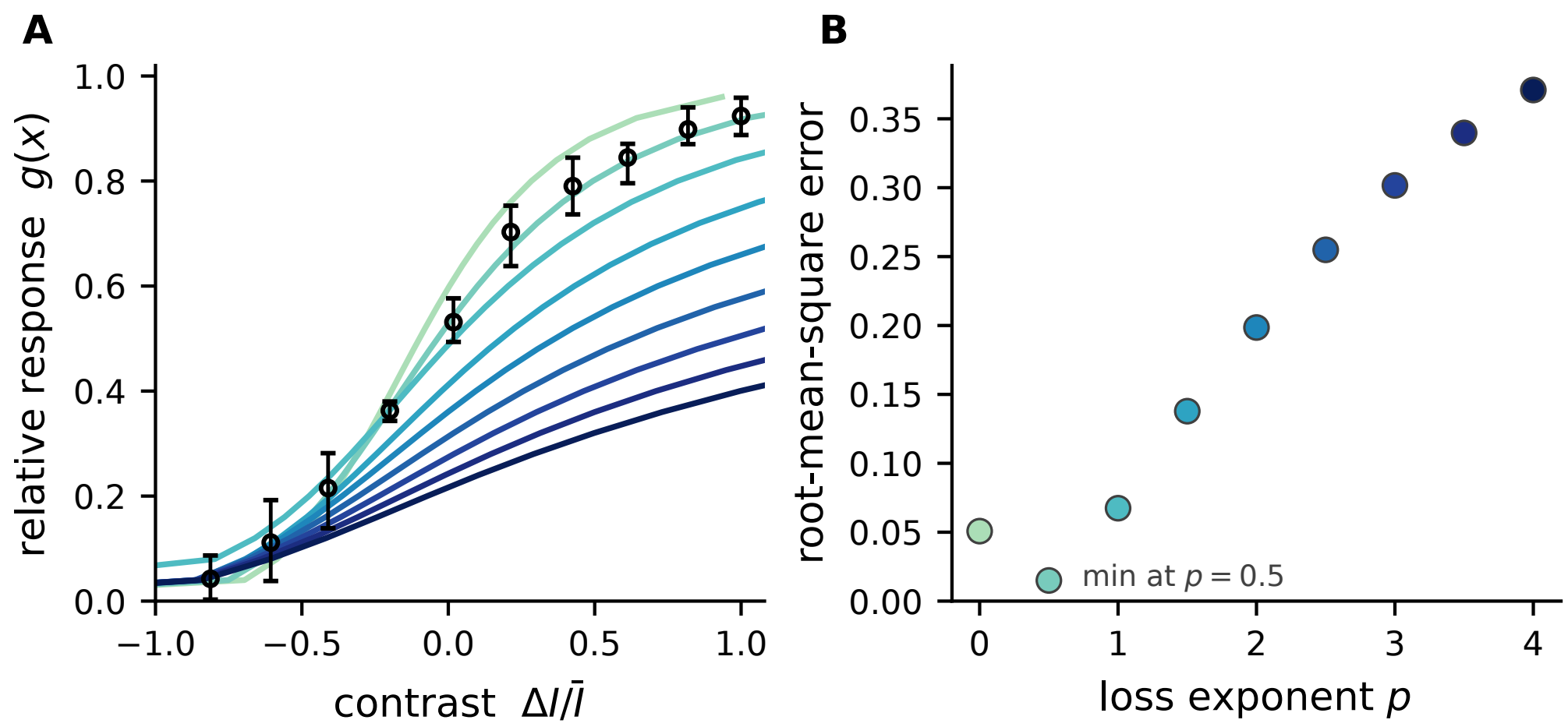

Fig C. The LMC response favors $p = \frac{1}{2}$ over infomax and larger loss exponents. **(A)** Optimal nonlinearities under L_p loss, computed from the fitted stimulus distribution and compared with the measured LMC response (circles, mean ± error bars). The $p = 0$ curve is the infomax solution; color encodes p. **(B)** Root-mean-square error (RMSE) between each predicted nonlinearity and the measured response. The RMSE falls from 0.051 under infomax to 0.015 at $p = \frac{1}{2}$, then increases monotonically for larger p.

6. Gray RM, Neuhoff DL. Quantization. IEEE Transactions on Information Theory. 1998;44(6):2325–2383. doi:10.1109/18.720541.
7. Zador P. Asymptotic quantization error of continuous signals and the quantization dimension. IEEE Transactions on Information Theory. 1982;28(2):139–149. doi:10.1109/tit.1982.1056490.
8. Laughlin SB. A simple coding procedure enhances a neuron's information capacity. Z Naturforsch. 1981;36:910–912.
9. Nadal JP, Parga N. Nonlinear neurons in the low-noise limit: a factorial code maximizes information transfer. Network: Computation in Neural Systems. 1994;5(4):565–581.
10. Lloyd S. Least squares quantization in PCM. IEEE Transactions on Information Theory. 1982;28(2):129–137. doi:10.1109/TIT.1982.1056489.
11. Max J. Quantizing for minimum distortion. IRE Transactions on Information Theory. 1960;6(1):7–12. doi:10.1109/TIT.1960.1057548.
12. van Steveninck RRdR, Lewen GD, Strong SP, Koberle R, Bialek W. Reproducibility and Variability in Neural Spike Trains. Science. 1997;275(5307):1805–1808.
13. Schilstra C, van Hateren JH. Blowfly Flight and Optic Flow. I. Thorax Kinematics and Flight Dynamics. Journal of Experimental Biology. 1999;202(11):1481–1490. doi:10.1242/jeb.202.11.1481.
14. Wang Z, Stocker AA, Lee DD. Efficient Neural Codes That Minimize Lp Reconstruction Error. Neural Computation. 2016;28(12):2656–2686.

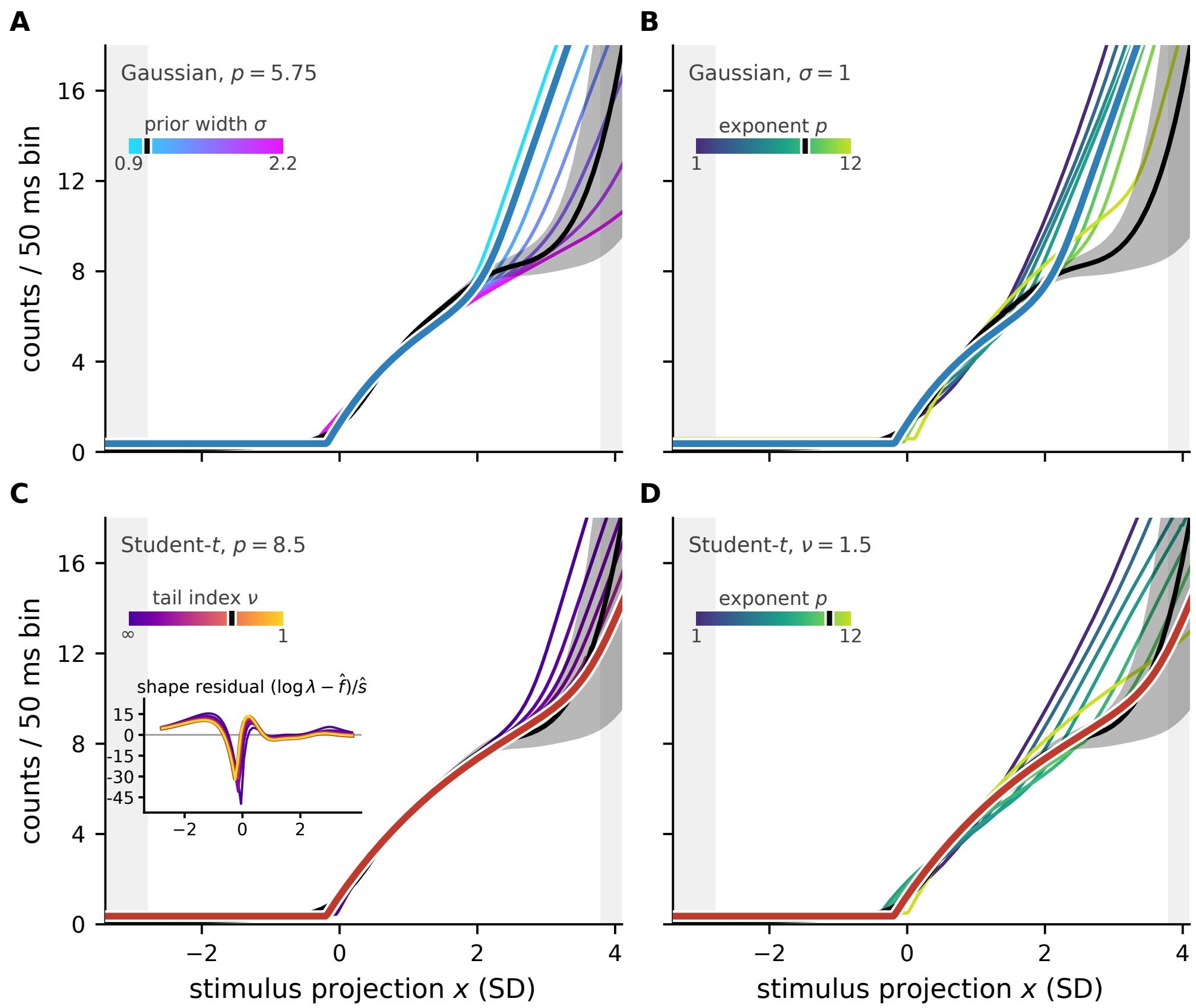

Fig D. The H1 nonlinearity favors a large loss exponent across Gaussian and heavy-tailed stimulus priors. Optimal LNP nonlinearities (colored curves) are compared with the measured H1 nonlinearity (black, posterior mean and approximate 95% band) after fitting their gain and baseline. Grey regions indicate stimulus values where the measured nonlinearity is poorly constrained. **(A)** Gaussian prior with its width σ varied at $p = 5.75$. **(B)** Standard normal prior with p varied. **(C)** Student-t prior with its tail index ν varied at $p = 8.5$; the inset shows the small differences among these solutions. **(D)** Student-t prior with $\nu = 1.5$ and p varied. In both prior families, changing p alters the predicted nonlinearity more strongly than changing the prior, and the closest match to H1 occurs at a large p, far from the infomax limit $p \to 0$.
